# Universal diffraction of atoms and molecules from a quantum reflection grating

**DOI:** 10.1126/sciadv.1500901

**Published:** 2016-03-18

**Authors:** Bum Suk Zhao, Weiqing Zhang, Wieland Schöllkopf

**Affiliations:** 1 Department of Chemistry, Ulsan National Institute of Science and Technology (UNIST), Ulsan 689-798, Korea.; 2 Department of Physics, UNIST, Ulsan 689-798, Korea.; 3 Fritz-Haber-Institut der Max-Planck-Gesellschaft, Faradayweg 4-6, 14195 Berlin, Germany.

**Keywords:** Quantum reflection, emerging beam resonance, helium dimer, matter-wave optics, grazing incidence atom optics, Rayleigh-Wood anomaly

## Abstract

Since de Broglie’s work on the wave nature of particles, various optical phenomena have been observed with matter waves of atoms and molecules. However, the analogy between classical and atom/molecule optics is not exact because of different dispersion relations. In addition, according to de Broglie’s formula, different combinations of particle mass and velocity can give the same de Broglie wavelength. As a result, even for identical wavelengths, different molecular properties such as electric polarizabilities, Casimir-Polder forces, and dissociation energies modify (and potentially suppress) the resulting matter-wave optical phenomena such as diffraction intensities or interference effects. We report on the universal behavior observed in matter-wave diffraction of He atoms and He_2_ and D_2_ molecules from a ruled grating. Clear evidence for emerging beam resonances is observed in the diffraction patterns, which are quantitatively the same for all three particles and only depend on the de Broglie wavelength. A model, combining secondary scattering and quantum reflection, permits us to trace the observed universal behavior back to the peculiar principles of quantum reflection.

## Teaser

Researchers observed universal diffraction of He, He_2_, and D_2_ quantum-reflected tens of nanometers above a ruled diffraction grating.

## INTRODUCTION

On the basis of the quantum-mechanical wave nature of particles, optical effects such as refraction, diffraction, and interferometry have been observed with “matter waves” of atoms, molecules, and more recently, clusters and macromolecules (*1*, *2*). In these experiments, unlike in classical optics with light, the interaction of the particle either with an external field or with the material of an optical element introduces a particle-dependent disturbance that, in general, tends to be detrimental for observing the optical effect of interest. For instance, diffraction patterns of atoms and molecules diffracted by a nanoscale transmission grating strongly depend on the van der Waals interaction between the particle and the grating material; an increase in interaction strength causes a narrowing of the effective width of the grating slits (*3*). Also, the diffraction peak intensities of He and D_2_ scattered from a crystal surface were found to strongly differ because of the different surface corrugations that result from different particle-solid interaction strengths (*4*). The effect of the molecule-surface interaction becomes severe for macromolecules and clusters, where it can result in a strong reduction of the fringe visibility as observed in matter-wave interferometry (*5*).

One possible way of overcoming this problem was recently demonstrated by Brand *et al*., who succeeded in using atomically thin nanoscale gratings made from a single-layer material such as graphene, thereby minimizing the particle-grating interaction (*6*). An alternative approach could be to use conventional diffraction gratings in such a way that the atoms or molecules do not come close to the solid surfaces, thereby strongly reducing the effects of the particle-grating interaction. Here, we demonstrate universal (viz., interaction-independent) diffraction by the *quantum reflection* of atoms and molecules from a conventional reflection grating. We have observed *emerging beam resonances* of identical shape for He atoms, D_2_ molecules, and even helium dimers, He_2_, under grazing incidence conditions. Coherent scattering of the particles results from quantum reflection from the long-range Casimir-Polder particle-surface potential tens of nanometers above the actual grating surface (*7*). By applying a secondary scattering model, we show how universal diffraction results from the peculiar principles governing quantum reflection. Here, universal diffraction means that the diffraction phenomena, including both angles and relative intensities of the diffraction peaks, depend solely on the de Broglie wavelength λ and are independent of the different strengths of the specific particle-grating interaction.

When an atom or molecule approaches a solid surface, it is exposed to the long-range attractive Casimir-Polder particle-surface interaction potential. In a classical description, this results in an acceleration of the particle toward the surface where, at the classical turning point, it will scatter back from the steep repulsive inner branch of the particle-surface potential. However, if the particle’s incident velocity is sufficiently small, the classical picture needs to be replaced by a quantum mechanical description. According to quantum mechanics, the particle’s de Broglie wavelength will vary along the slope of the attractive Casimir-Polder potential. If the length of the slope appears to be short on a length scale set by the de Broglie wavelength, the attractive potential effectively acts as an impedance discontinuity to the particle’s wave function. As a result, there is a detectable probability for the wave function to be quantum-reflected at the Casimir-Polder potential, way in front of the actual surface (*8*–*11*). In the limit of vanishing incident particle velocity (corresponding to infinite incident wavelength), the Casimir-Polder potential effectively resembles a step in the potential, and thus, the probability for quantum reflection approaches unity.

Quantum reflection from a solid has been observed with ultracold, metastable atoms (*12*, *13*), atom beams (*14*–*16*), and even Bose-Einstein condensates (*17*, *18*). Recently, coherent and nondestructive quantum reflection from a diffraction grating was reported for the van der Waals clusters He_2_ (*7*) and He_3_ (*19*). The exceptionally small binding energies of 10^−7^ eV (He_2_) and 10^−5^ eV (He_3_) are orders of magnitude smaller than the cluster-surface potential well depth of 10^−2^ eV. However, because dimers and trimers are quantum-reflected tens of nanometers above the surface, they do not come close to where surface-induced forces would inevitably break up the fragile bonds.

Emerging beam resonance, also known as the Rayleigh-Wood anomaly, is a phenomenon that occurs in grating diffraction when conditions (wavelength, grating period, and incidence angle) are such that a diffracted beam of order *m* emerges from the grating plane. For instance, when, for given wavelength and grating period, the incidence angle is continuously varied from grazing toward normal incidence, the *m*th-order diffraction beam will, at some point, change from an evanescent wave state (pre-emergence) to emerging and, eventually, to a freely propagating wave above the grating plane (post-emergence). The incidence angle at which the emergence occurs is referred to as the *m*th-order Rayleigh angle. The emergence of a new beam causes abrupt intensity variations of the other diffraction beams marking the emerging beam resonance. The effect was first observed with visible light by Wood (*20*) and explained by Rayleigh in 1907 (*21*). Only recently was it observed in atom diffraction (*22*). Here, we report evidence for the emerging beam resonance effect for the helium dimer.

## RESULTS

**Fig. 1:**
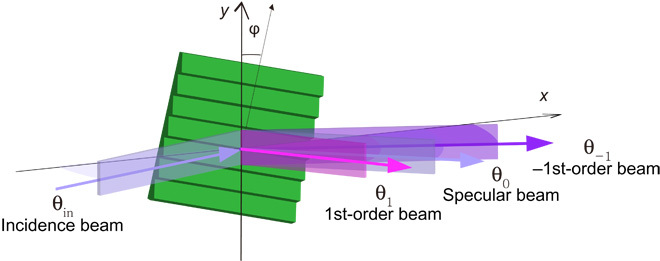
Schematic representation of the experimental setup. A collimated atomic or molecular beam is quantum-reflected from the diffraction grating. The figure is not to scale: The actual incidence angles and diffraction angles, which are defined with respect to the grating plane, are on the order of 1 mrad only. As shown here, the azimuth angle φ is chosen such that the facet surfaces are tilted away from the source, leading to a blazing of the negative-order diffraction beams. Out-of-plane diffraction effects have been neglected in this figure for the sake of simplicity. A detailed description of the apparatus is provided in the Supplementary Materials.

**Fig. 2:**
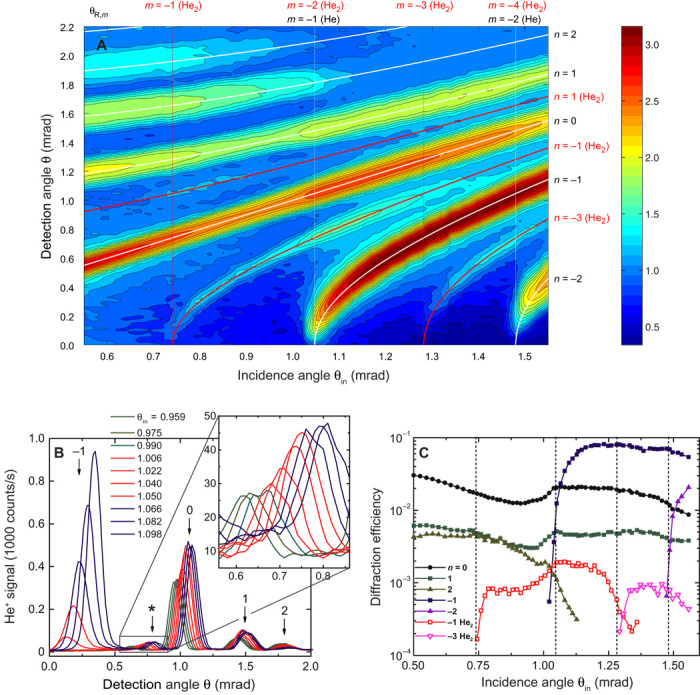
Diffraction data for helium atoms and dimers. (**A**) Two-dimensional contour plot of the He^+^ signal from 64 diffraction spectra measured for φ = −33.5 mrad and incidence angles from θ_in_ = 0.5 to 1.56 mrad as a function of incidence and detection angles. The signal is plotted on a logarithmic contour scale ranging from 10^0.5^ to 10^3^ counts/s. White solid lines indicate the calculated *n*th-order diffraction angles of helium atoms, which are identical to the (2*n*)th-order diffraction angles of helium dimers. Red solid lines represent the calculated odd-order [(2*n* − 1)th] diffraction angles of dimers, which do not coincide with diffraction angles of atoms. The vertical lines indicate the calculated Rayleigh incidence angles θ_R,*m*_ corresponding to the emergence of the *m*th-order diffraction peaks of atoms and dimers as indicated on top of the plot. (**B**) Diffraction patterns at 10 different incidence angles in the vicinity of the Rayleigh incidence angle θ_R,−1_(He) = θ_R,−2_(He_2_) = 1.047 mrad, where the –1st- and –2nd-order peaks of the monomer and dimer emerge, respectively. The numbers on top of the arrows indicate the orders of the atomic diffraction peaks. The asterisk marks the –1st-order peak of the dimer, which is shown enlarged in the inset. (**C**) Diffraction efficiencies (as defined in Materials and Methods) as a function of incidence angle. The dashed vertical lines indicate the same Rayleigh incidence angles as in (A).

A schematic of the experimental setup is shown in Fig. 1. The 20-μm-period echelette grating is described in Materials and Methods, whereas further details of the apparatus are provided in the Supplementary Materials. Diffraction data observed with a helium beam, containing both atoms and dimers at de Broglie wavelengths of 0.33 and 0.16 nm, respectively, are shown in Fig. 2A for a range of incidence and diffraction angles. The –1st-order diffraction peak of helium atoms, which emerges at an incidence angle of θ_in_ = 1.047 mrad, shows the strongest overall signal, larger than the specular (0th-order) beam. The –2nd-order diffraction beam, emerging at θ_in_ = 1.480 mrad, and the first-order beam of the atoms are clearly visible. Higher-order (*n* = 2 and *n* = 3) atomic diffraction beams show up with less intensity decaying with increasing incidence angle. In addition, –1st- and –3rd-order peaks of He_2_ are clearly visible for incidence angles larger than 0.75 and 1.3 mrad, respectively (*7*). Furthermore, for incidence angles from 0.7 to 0.9 mrad, a weak signal of the dimer’s first-order peak appears. For both atoms and dimers, the larger intensities of negative-order peaks result from the grating blaze (*23*). At Rayleigh angles, indicated in Fig. 2A, diffraction peaks appear at grazing emergence, with their intensities steeply increasing with incidence angle.

Figure 2B shows angular spectra (corresponding to cross sections of the contour plot along the *y* axis) for incidence angles around the –1st-order Rayleigh angle θ_R,−1_(He) = 1.04 mrad, where the monomer’s –1st-order peak and the dimer’s –2nd-order peak emerge. For θ_in_ ≤ 0.99 mrad, the –1st-order diffraction beam has not yet appeared (pre-emergence); there is no peak at angles θ ≤ 0.5 mrad (greenish traces in Fig. 2B). In this regime, the specular and first-order peaks of the atoms as well as the –1st-order peak of the dimers (inset in Fig. 2B) show little or no change with incidence angle.

For incidence angles in the range θ_in_ = 1.0 to 1.05 mrad, the new diffraction peak is emerging progressively at θ ≤ 0.5 mrad (red traces in Fig. 2B). Because of a finite incident beam divergence of about 50 μrad, the emergence of the new peak does not occur at a well-defined incidence angle but rather is spread out over an interval of angles (*23*). This is reflected by the fact that for θ_in_ = 1.040 and 1.050 mrad, the partly emerged peaks share the left slope (*24*). Concurrent to the emergence of a new peak, the specular peak and the first-order peak of the atoms exhibit a steep increase from 340 to 500 counts/s and from 70 to 105 counts/s, respectively. An even stronger increase of about 100% is found for the –1st-order diffraction peak of the helium dimers, as can be seen in the inset of Fig. 2B. We interpret these rather abrupt intensity variations as a manifestation of the emerging beam resonance effect for He and He_2_ upon the emergence of the –1st- and –2nd-order peak, respectively. For incidence angles θ_in_ ≥ 1.066 mrad, the new diffraction beam appears fully emerged from the grating (post-emergence; bluish traces in Fig. 2B).

Figure 2C shows diffraction efficiencies analyzed from the data shown in Fig. 2A. It is evident in the graph that at θ_R,−1_(He) = θ_R,−2_(He_2_) = 1.04 mrad, the diffraction efficiencies not only for He (*n* = 0 and 1) but also for He_2_ (*n* = −1) exhibit cusps characteristic for the emerging beam resonance effect (*22*). In addition, when the dimer –3rd-order beam emerges at θ_R,−3_(He_2_) = 1.28 mrad, the dimer –1st-order diffraction efficiency exhibits a rapid decrease.

## DISCUSSION

**Fig. 3:**
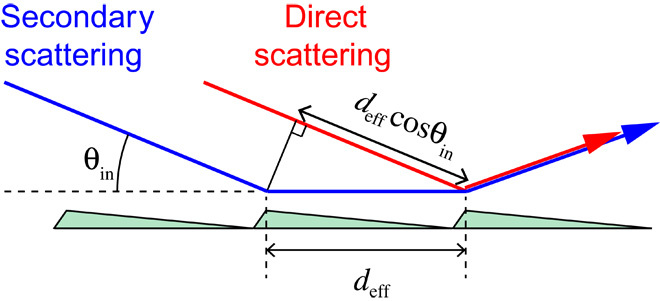
Direct scattering and secondary scattering from neighboring grating units. The red arrow indicates direct scattering: the scattering of the incident beam from a grating unit. In addition, we consider secondary scattering as visualized by the blue arrow; the incident beam first scatters off a grating unit and propagates parallel to the grating surface plane for a distance *d*_eff_, until it scatters off from the neighboring grating unit.

To analyze the emerging beam resonance behavior, we apply the multiple scattering model introduced by Rayleigh (*21*) and Fano (*25*, *26*). As depicted in Fig. 3, the *n*th-order diffraction beam amplitude *A*_*n*_ is approximated as the constructive interference of direct and secondary scattering waves; *A*_*n*_ = *A*_*n*_^(1)^ + *A*_*n*_^(2)^. For θ_in_ = θ_R,*m*_, the geometrical path length difference between direct and secondary scattering, *d*_eff_ (1 − cosθ_in_), is equal to |*m*|λ, giving rise to fully constructive interference. An additional phase shift Φ is induced by the particle-surface interaction potential for an atom or molecule propagating along the path of length *d*_eff_ between the first and second scattering occurrences (Fig. 3). For quantum reflection under Rayleigh conditions, one finds Φ = −*m* π [1 + cosθ_R,*m*_] ≈ −*m* 2π (see Materials and Methods), which also corresponds to fully constructive interference.

Although this simple model cannot account for the detailed shape of the emerging beam resonance effect displayed in Fig. 2C, it allows us to derive two main aspects. First, under Rayleigh conditions, we expect constructive interference between direct and secondary scattering. Thus, the emergence of an *m*th-order beam is expected to increase the other diffraction peaks, including the specular peak, the more so the more intense the emerging beam is. Consequently, the overall reflectivity shows a steep variation under Rayleigh conditions, which is in full agreement with experimental results (*22*). Second, the calculated phase shift depends on the de Broglie wavelength as the sole parameter. Hence, at a given de Broglie wavelength, the model predicts the same (universal) behavior for any atom or molecule. This comes as a surprise because quantum reflection is inherently linked with the Casimir-Polder potential, which is particle-specific.

The derivation of the phase shift Φ is based on two assumptions (see Materials and Methods). The particle-surface potential probed by the particle along its path between the first and second scattering occurrences is (i) constant and (ii) equal to the particle’s incident perpendicular kinetic energy, that is, the energy associated with the incident velocity component perpendicular to the grating plane. The second assumption is an approximation that follows from the principles of quantum reflection (*9*–*11*). It holds independent of the specific particle properties. As a result, different atoms or molecules at the same de Broglie wavelength will be quantum-reflected at different heights above the surface, but their wave functions will acquire the same phase shift Φ. This peculiarity of quantum reflection is the origin of universal (interaction-independent) diffraction.

To check this prediction of universal behavior, we repeated the experiment with He and D_2_ under conditions such that their de Broglie wavelengths are identical to the wavelength of He_2_ in the data shown in Fig. 2. All other experimental parameters were kept unchanged.

**Fig. 4:**
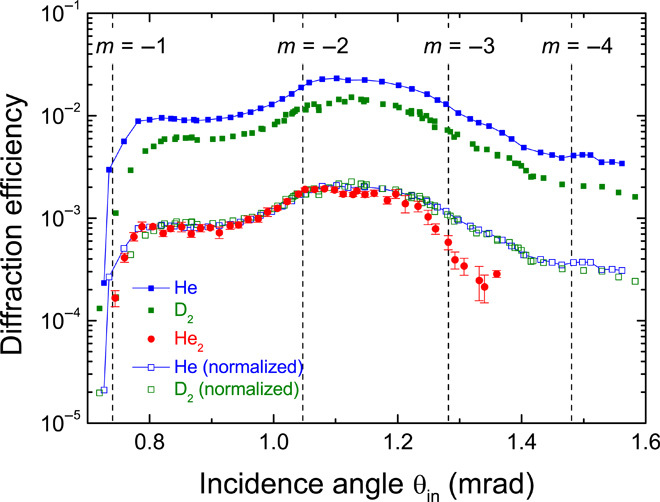
Comparison of the –1st-order diffraction efficiency curves for He, He_2_, and D_2_ at corresponding conditions. The He_2_ data are replotted from Fig. 2C. The He and D_2_ data were collected for *T*_0_ = 35 K and *P*_0_ = 7.3 bar (He) and 0.8 bar (D_2_) for otherwise identical machine parameters. Under these conditions, the de Broglie wavelengths of He and D_2_ are 0.17 nm, which is identical to the dimer’s wavelength. The vertical lines indicate Rayleigh angles of incidence for the emergence of *m*th-order diffraction peaks. As a result of identical de Broglie wavelengths, the Rayleigh angles are the same for all three species. To account for differences in the overall quantum reflection probabilities, the He and D_2_ data were normalized by factors of 0.09 and 0.15, respectively.

Figure 4 shows a direct comparison of the –1st-order diffraction efficiency curves. We find excellent agreement of the data for the three species (except for incident angles larger than about 1.25 mrad), thereby confirming the prediction of universal diffraction. At larger incident angles, He and D_2_ diffraction efficiencies still overlap, but the He_2_ efficiency is found to taper off. A possible explanation for this deviation could be that some dimers start to break up as they approach closer to the surface with increasing incidence angle. Furthermore, we note that universal behavior was also found for the –3rd-order diffraction efficiency curves of He, He_2_, and D_2_.

In conclusion, we have observed emerging beam resonances for He, He_2_, and D_2_ quantum-reflected from an echelette diffraction grating at grazing incidence. Our observation indicates that He_2_, despite its fragile bond, can undergo double coherent, nondestructive scattering; under Rayleigh conditions, dimers scattered at a grating unit propagate parallel to the surface, scatter a second time at another grating unit without breakup, and interfere with directly scattered dimers.

Furthermore, a simple approximate calculation of the relative phase between the direct and the secondary scattering paths indicates constructive interference under Rayleigh conditions independent of the particle-specific Casimir-Polder interaction with the grating. Diffraction data of He, He_2_, and D_2_ under conditions of identical de Broglie wavelength confirm this universal behavior. Because the effect is independent of the particle-specific properties, universal diffraction from a quantum-reflection grating can, in principle, be applied to larger molecules as well. The only prerequisite is the preparation of a sufficiently large de Broglie wavelength corresponding to the velocity component perpendicular to the grating plane, thereby providing a sufficient quantum reflection probability. In future experiments, this could possibly be achieved by applying a state-of-the-art molecular-beam deceleration technique (*27*) or by choosing an even smaller incidence angle than the ones shown here.

## MATERIALS AND METHODS

### Diffraction grating

The commercial plane ruled echelette grating (Newport 20RG050-600-1; period *d* = 20 μm; blaze angle, 14 mrad) is aligned in a conical mount (*28*); the grooves are almost parallel to the incidence plane. We define the azimuth angle φ as the angle between the grooves and incidence plane. Here, a negative azimuth angle was chosen to enhance the intensities of emerging diffraction beams (*22*). The exact value of φ is determined from fitting the diffraction angle curves shown in Fig. 2A. An agreement between the lines and the positions of the observed peaks is found for φ = −33.5 mrad corresponding to *d*_eff_ = 597 μm.

### Rayleigh angles

The *n*th-order diffraction angle θ_*n*_ can be calculated by the approximated grating equation for conical diffraction, cosθ_in_ − cosθ_*n*_ = *n*(λ/*d*_eff_) with effective period *d*_eff_ = *d*/|sinφ| (*23*). The Rayleigh angle θ_R,*m*_ is derived by inserting cosθ_R,*m*_ − 1 = *m*λ/*d*_eff_ into the grating equation. Because the de Broglie wavelength of a particle is inversely proportional to its mass, Rayleigh angles for monomers and dimers at the same particle velocity follow a simple relationship: θ_R,*m*_(He) = θ_R,2*m*_(He_2_). For instance, in the measurements shown in Fig. 2, the de Broglie wavelength λ is 0.327 nm for He and 0.164 nm for He_2_, resulting in Rayleigh angles θ_R,−1_(He) = θ_R,−2_(He_2_) = 1.047 mrad.

### Diffraction efficiencies

We define the diffraction efficiency of an *n*th-order peak as the ratio of its area to the incidence beam area. Diffraction peak areas are determined by fitting each peak of an individual diffraction pattern, like the ones shown in Fig. 2B, with a Gaussian. The incidence beam area is determined from an angular spectrum measured with the grating removed from the beam path. The incident beam signal is dominated by the atomic beam component with just a small contribution of a few percent due to helium dimers. Therefore, the normalization to the incident beam peak area results in a slight underestimation of the actual diffraction efficiencies (given as the ratio of the *n*th-order beam intensity to the incident beam intensity for either atoms or dimers) for the atoms and in a severe underestimation for the dimers. Thus, the diffraction efficiencies for dimers plotted in Fig. 2C should be considered as having arbitrary units and cannot be compared quantitatively to their atomic counterparts.

### Diffractive evanescent waves

Evanescent waves (*29*) contribute to *A*_*n*_^(2)^ because they propagate parallel to the grating surface plane. Evanescent waves result from diffraction into higher-order beams whose wave vector normal component is imaginary (that is, nonpropagating) (*26*, *29*). A diffraction beam of order *m* is freely propagating as long as the incidence angle is larger than the Rayleigh angle, θ_in_ > θ_R,*m*_; it is emerging under the Rayleigh condition, θ_in_ = θ_R,*m*_; and it is evanescent for θ_in_ < θ_R,*m*_. Evanescent waves contribute to *A*_*n*_^(2)^ in the secondary scattering model. Close to the Rayleigh angle, θ_in_ ≤ θ_R,*m*_, a significant contribution of the *m*th-order evanescent wave to *A*_*n*_^(2)^ can be expected (*26*). This is why the emerging beam resonance can, potentially, affect the other diffraction peak intensities already in the pre-emergence regime at θ_in_ ≤ θ_R,*m*_ (*23*).

### Quantum reflection

Quantum reflection of a particle from the attractive particle-surface potential takes place at a range of heights above the surface, where the reflection probability is nonzero. As a rule of thumb, this range of heights is around the location where the kinetic energy associated with the velocity normal component is equal to the absolute magnitude of the attractive particle-surface interaction potential (*9*–*11*). (See the Supplementary Materials for information on how the rule of thumb is derived.)

The attractive part of the potential can be approximated by a Casimir-Polder surface potential, *V*(*z*) = −*C*_3_*l*/ [(*l* + *z*)*z*^3^]. Here, *z* denotes the distance from the surface, and the product of the van der Waals coefficient *C*_3_ and a characteristic length *l* (*l* = 9.3 nm for He) indicates the transition from the van der Waals (*z* << *l*) to the retarded Casimir-Polder regime (*z* >> *l*) (*11*).

For He_2_, one expects *C*_3_ to be two times larger and *l* to be the same as compared to the He atom, because the extremely weak van der Waals bond of the dimer is too feeble to cause a significant disturbance to the electron shells of the two He atoms that, thus, can be treated as separate atoms (*30*). Because He and He_2_, coexisting in a helium beam, have the same velocity, the dimer’s kinetic energy is twice that of the atoms. As a result, at a given incidence angle, He and He_2_ in a beam are quantum-reflected at about the same distance from the surface, because the increased incidence energy of He_2_ is compensated for by its larger *C*_3_ coefficient.

Using *C*_3_ = 0.202 meV nm^3^ between helium and aluminum (*31*), at θ_in_ = 0.740, 1.047, 1.282, and 1.480 mrad, which are the Rayleigh angles for the emergence of the –1st- to –4th-order He_2_ peaks in Fig. 2, the surface distance, where quantum reflection takes place, is estimated from the rule of thumb to be 35.3, 29.2, 26.2, and 24.2 nm, respectively.

However, for identical de Broglie wavelengths, as in Fig. 4, quantum reflection of the three species is expected to occur at different heights above the surface. This can easily be seen by considering the rule of thumb and the strength of the particle-surface interaction, which is stronger for D_2_ than for He. As to He and He_2_, for identical de Broglie wavelengths, the dimer’s incident kinetic energy is just one-half of the monomer’s kinetic energy. Therefore, quantum reflection of He_2_ is expected to occur at larger distances above the grating surface as compared to He.

### Secondary scattering phase shift

It is straightforward to calculate the additional phase shift Φ induced by the particle-surface potential for an atom or molecule of mass *M* and incident kinetic energy *E* propagating along the path *d*_eff_ between the first and second scattering (see Fig. 3). We apply the rule of thumb stating that quantum reflection takes place at about that distance to the surface where the particle’s incident kinetic energy (corresponding to the motion along the surface-normal coordinate) equals the absolute magnitude of the Casimir-Polder potential energy (*9*–*11*). Therefore, for secondary scattering, we can approximate the potential energy probed by the particle along the additional path of length *d*_eff_ to be equal to *E*_perp_ = (1/2)*Mv*_perp_^2^. Here, *v*_perp_ denotes the normal component of the incident particle velocity. The particle-surface potential–induced phase shift can be calculated as Φ = (*k* − *k*_0_)*d*_eff_ = $\frac{\sqrt{2M}}{\hbar }d_{\text{eff}}[\sqrt{E+E_{\text{perp}}}-\sqrt{E}]$ ≈ $\frac{\sqrt{2M}}{\hbar }d_{\text{eff}}\frac{1}{2}\sqrt{E}{\text{sin}}^{2}\theta_{\text{in}}$ = $\frac{1}{2}k_{0}d_{\text{eff}}{\text{sin}}^{2}\theta_{\text{in}}$, where the square root $\sqrt{E+E_{\text{perp}}}$ has been approximated by its Taylor expansion justified by *E*_perp_ << *E*. *k* and *k*_0_ denote the particle’s wave vector in the presence and absence of the particle-surface interaction potential, respectively. In the former case, the kinetic energy is increased by the potential energy of the atom-surface interaction. We assume *k* to be constant along the path *d*_eff_ between the first and second scattering, corresponding to a constant height of the particle above the surface of the grating facet.

The phase shift Φ = $\frac{\pi }{\lambda }d_{\text{eff}}{\text{sin}}^{2}\theta_{\text{in}}$ can be further simplified. For Rayleigh conditions of *m*th-order emergence, given by cosθ_R,*m*_ − 1 = *m*λ/*d*_eff_, we get sin^2^θ_R,*m*_ = (1 + cosθ_R,*m*_)(1 − cosθ_R,*m*_) = (1 + cosθ_R,*m*_) $\frac{-m\lambda }{d_{\text{eff}}}$. As a result, one finds that, for quantum reflection under Rayleigh conditions, the phase shift induced by the potential is Φ = −*m* π [1 + cosθ_R,*m*_] ≈ −*m* 2π.

## Supplementary Material

http://advances.sciencemag.org/cgi/content/full/2/3/e1500901/DC1

## SUPPLEMENTARY MATERIALS

Supplementary material for this article is available at http://advances.sciencemag.org/cgi/content/full/2/3/e1500901/DC1 Source and helium beam. Slits, apparatus geometry, and definition of angles. Mass spectrometer detector and apparatus resolution. Derivation of the “rule of thumb” of quantum reflection. Fig. S1. Schematic of the quantum-reflection diffraction setup. References (*32*, *33*)
