# Supplementary material for: Universal diffraction of atoms and molecules from a quantum reflection grating
Source: Sci Adv. 2016 Mar 18;2(3):e1500901. doi: 10.1126/sciadv.1500901 (PMC4803490; doi:10.1126/sciadv.1500901)
Supplement: http://advances.sciencemag.org/cgi/content/full/2/3/e1500901/DC1 [file supp_2_3_e1500901__index.html]

Science Advances | Science Advances

## Supplementary Materials

**This PDF file includes:**

- Source and helium beam.
- Slits, apparatus geometry, and definition of angles.
- Mass spectrometer detector and apparatus resolution.
- Derivation of the “rule of thumb” of quantum reflection.
- Fig. S1. Schematic of the quantum-reflection diffraction setup.
- References (*32, 33*)

Download PDF

**Files in this Data Supplement:**

- Adobe PDF - 1500901\_SM.pdf
