# Supplementary material for: Universal diffraction of atoms and molecules from a quantum reflection grating
Source: Sci Adv. 2016 Mar 18;2(3):e1500901. doi: 10.1126/sciadv.1500901 (PMC4803490; doi:10.1126/sciadv.1500901)
Supplement: http://advances.sciencemag.org/cgi/content/full/2/3/e1500901/DC1 [file 1500901_SM.pdf]

Science Advances

AAAS

advances.sciencemag.org/cgi/content/full/2/3/e1500901/DC1

Supplementary Materials for

Universal diffraction of atoms and molecules from a quantum reflection grating

Bum Suk Zhao, Weiqing Zhang, Wieland Schöllkopf

Published 18 March 2016, *Sci. Adv.* **2**, e1500901 (2016)
DOI: 10.1126/sciadv.1500901

The PDF file includes:

Source and helium beam.
Slits, apparatus geometry, and definition of angles.
Mass spectrometer detector and apparatus resolution.
Derivation of the "rule of thumb" of quantum reflection.
Fig. S1. Schematic of the quantum-reflection diffraction setup.
References (*32*, *33*)

Supplementary Materials

Source and helium beam

The helium-beam diffraction apparatus used in this work has been described in detail before (*7, 19, 23*). The setup is shown schematically in Fig. S1. It combines a cryogenic helium atom and cluster beam source with ultra-high angular resolution grating diffraction. The helium beam is formed by free-jet expansion of pure ^{4}He gas from a source cell through a 5-μm-diameter orifice into a vacuum of $\approx 10^{-6}$ mbar. The stagnation temperature and pressure of the gas in the source cell is T_0 = 9.0 K and P_0 = 1 bar, respectively. Under these conditions the helium gas cools down in the supersonic adiabatic expansion to below 1 mK resulting in the formation of helium dimers as well as negligible amounts of trimers and larger clusters *(32)*. The low temperature corresponds to a narrow velocity distribution of the helium beam characterized by a most probable velocity measured to be 305 m/s. At this velocity the de Broglie wavelength λ is 0.327 and 0.164 nm for He and He_2, respectively. In a second set of measurements a He atom beam and a D_2 molecular beam were made at T_0 = 35 K and P_0 = 7.3 bar (He) and 0.8 bar (D_2). At these conditions the de Broglie wavelengths of He and D_2 are also 0.164 nm giving corresponding conditions for all three particles.

Slits, apparatus geometry, and definition of angles

After passing a skimmer, the atom beam is collimated by two vertical slits (20 μm wide, 3 mm high) separated by 1 m along the horizontal beam direction. This results in a divergence of merely 50 μrad of the beam incident at grazing incidence onto the diffraction grating positioned 0.4 m downstream from the second slit. The incidence angle θ_{in} is measured with respect to the grating surface. It can be varied by rotating the grating around the *y* axis (vertical) that passes through the center of the grating surface and is perpendicular to the incidence plane (horizontal). The latter is formed by the grating normal and the incident beam direction. The helium beam scattered from the diffraction grating passes through a

detector-entrance slit (25 μm wide, 5 mm high) located 0.38 m downstream from the grating, thereby defining the angular resolution of the detector to be 66 μrad.

Mass spectrometer detector and apparatus resolution

In the detector the neutral He atoms and dimers are ionized in collisions with electrons of 120 eV energy. The resulting He^+ ion, which is readily formed by fragmentation of the weakly bound neutral dimer *(33)*, is mass selected and detected in a non-commercial magnetic-sector mass spectrometer. The detection angle θ, defined with respect to the grating surface, is adjusted by precisely rotating the detector around the y axis. The effective angular resolution of the apparatus results from the angular resolution of the detector convoluted with the incidence beam divergence and is less than 100 μrad, sufficient to separate the diffraction peaks of the dimer from those of the monomer. Diffraction patterns at a given incidence angle are obtained by rotating the detector in small angular steps of 20 μrad and counting the He^+ signal.

Derivation of the "rule of thumb" of quantum reflection

The derivation of the phase shift Φ is based on the assumption that quantum reflection takes place at about a distance z_0 above the surface where the particle's incident kinetic energy (corresponding to the motion along the surface-normal coordinate) $E_{perp} = (1/2)Mv_{perp}^2$ equals the absolute magnitude of the Casimir-Polder potential energy (*9–11*)

$$|V(z_0)| = E_{perp} \qquad \text{Eq. (1)}$$

Here M and v_{perp} denote the particle mass and the normal component of the incident particle velocity, respectively.

In theoretical studies quantum reflection was found to occur in a region of particle-to-surface distance z where the Wentzel-Kramers-Brillouin (WKB) approximation breaks down *(8-11)*. WKB breakdown occurs where the potential energy changes significantly across the particle's local de Broglie wavelength. This can be expressed as a violation of the WKB approximation criterion

$$\frac{1}{k^2}\frac{dk}{dz} = \frac{\hbar}{p^2}\frac{dp}{dz} << 1 \qquad \text{Eq. (2)}$$

Mody *et al. (10)* (Eqs. (48) to (52)) as well as Friedrich *et al. (11)* (Eqs. (A4) and (A5)) calculate where the strongest violation of Eq. (2), corresponding to a maximum of the left hand side of Eq. (2), occurs for potentials of the form $V(z) = -C_n / z^n$ with $n = 3$ or 4. Their calculations reveal that this occurs approximately at the particle-to-surface distance z_0 defined by Eq. (1). Furthermore, Friedrich *et al. (11)* (Appendix) lay out that Eq. (2) is just an approximation to the WKB criterion which, in its exact form, reads

$$\hbar^2\left(\frac{3}{4}\frac{(p')^2}{p^4} - \frac{1}{2}\frac{p''}{p^3}\right) << 1 \qquad \text{Eq. (3)}$$

These authors show that, for a potential of the form $V(z) = -C_n / z^n$ with $n = 4$, the maximum violation of Eq. (3) occurs right at the distance z_0 defined by Eq. (1), and they further show that this, indeed, corresponds to the position where quantum reflection is most likely to occur.

Note that the rule of thumb for z_0 as expressed by Eq. (1), although it does not explicitly contain the gradient of the potential $V(z)$, has been derived from the condition that the potential varies significantly over a de Broglie wavelength (violation of the WKB-criteria Eqs. (2) and (3)).

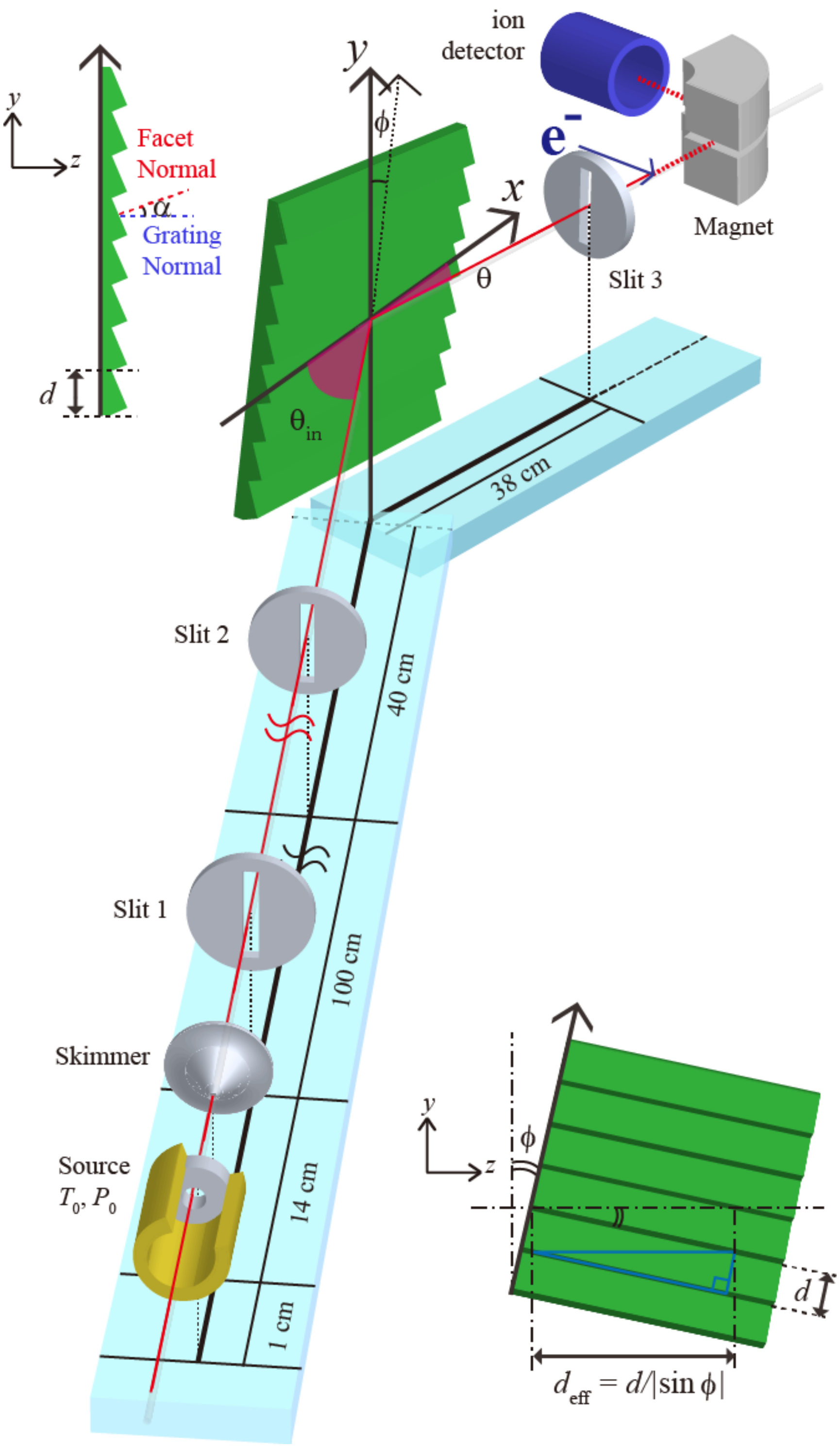

Fig. S1. Schematic of the quantum-reflection diffraction setup. An atomic or molecular beam of either helium or deuterium is formed by expansion of the pure gas (temperature T_0, pressure P_0) through a pinhole aperture into vacuum. The collimated beam is quantum reflected from the diffraction grating

in grazing incidence geometry. The incidence and detection angles θ_{in} and θ (both on the order of 1 mrad) are defined with respect to the grating surface plane. The commercial blazed echelette grating is aligned in conical mount with the grooves almost parallel to the incident beam axis. Definitions of the grating blaze angle α, azimuth angle ϕ as well as the grating period d and effective grating period d_{eff} are indicated. Angles and dimensions are not drawn to scale for the sake of visibility. Downstream of slit 3, in the detector, neutral atoms and molecules are ionized by electron impact with subsequent magnetic mass selection and detection of the ions.
